# Diagnostic delay in patients with giant cell arteritis: results of a fast-track clinic

**DOI:** 10.1007/s10067-023-06739-w

**Published:** 2023-08-31

**Authors:** Marieke van Nieuwland, Edgar M. Colin, Dennis Boumans, Marloes Vermeer, Elisabeth Brouwer, Celina Alves

**Affiliations:** 1 Department of Rheumatology and Clinical Immunology, Ziekenhuisgroep Twente (Hospital Group Twente), Almelo, The Netherlands; 2 Department of Rheumatology and Clinical Immunology, University of Groningen, University Medical Center Groningen, Groningen, The Netherlands; 3 ZGT Academy, Ziekenhuisgroep Twente (Hospital Group Twente), Almelo, The Netherlands

**Keywords:** Delayed diagnosis, Epidemiology, Giant cell arteritis, Referral and consultation, Vasculitis

## Abstract

Giant cell arteritis (GCA) can lead to severe complications if left untreated. The aim of this study was to describe time from onset of symptoms to diagnosis and treatment in GCA suspected patients in a fast-track clinic (FTC), and secondarily to assess the influence of GCA symptoms on this time. A retrospective cohort consisting of suspected GCA patients who visited the FTC between January 2017 and October 2019 was used. Time between symptom onset, first general practitioner visit, FTC referral, first FTC visit, and treatment initiation was analysed. Furthermore, this was stratified for subtypes of GCA and GCA symptoms. Of 205 patients referred with suspected GCA, 61 patients received a final diagnosis of GCA (GCA+) and 144 patients had no GCA (GCA−). Median time after onset of symptoms to first FTC visit was 31.0 days (IQR 13.0–108.8) in all referred patients. Time between onset of symptoms and first GP visit was 10.5 (4.0–36.3) days, and time between first GP visit and FTC referral was 10.0 (1.0–47.5) days. Patients were generally seen at the FTC within 1 day after referral. For patients with isolated cranial GCA (*n* = 41), median delay from onset of symptoms to treatment initiation was 21.0 days (11.0–73.5), while this was 57.0 days (33.0–105.0) in patients with extracranial large-vessel involvement (*n* = 20) (*p* = 0.02). Our results indicate considerable delay between symptom onset and FTC referral in patients suspected of GCA. Suspected patients were examined and GCA+ patients were treated instantly after referral.

**Table:** 

**Key Points**
• *GCA can cause severe complications with delayed treatment, but non-specific symptoms make diagnosis challenging.* • *Diagnostic delay still occurs despite introducing a successful fast-track clinic resulting from delay between start of symptoms and FTC referral.* • *Patients who presented with constitutional symptoms had longer delay than patients who presented with isolated cranial symptoms.*

• *GCA can cause severe complications with delayed treatment, but non-specific symptoms make diagnosis challenging.*

• *Diagnostic delay still occurs despite introducing a successful fast-track clinic resulting from delay between start of symptoms and FTC referral.*

• *Patients who presented with constitutional symptoms had longer delay than patients who presented with isolated cranial symptoms.*

## Introduction

Giant cell arteritis (GCA) is the most common systemic vasculitis and can lead to severe complications when left untreated. GCA mostly occurs in patients over 50 years, peaking between ages 70 and 80 [1]. The incidence is relatively low (10 per 100,000 person-years) [2]. GCA is classically known for inflammation of cranial arteries (cranial GCA (C-GCA)), including the temporal arteries; however, its disease spectrum also includes an extracranial phenotype referred to as large-vessel GCA (LV-GCA) [3, 4]. Inflammation-induced ischemia may cause new headache, visual symptoms, jaw claudication, and scalp tenderness; however, more general and atypical symptoms are also common [4].

GCA is a medical emergency as it can cause severe complications such as stroke, permanent vision loss, and aneurysms. Complications can arise within days and can be prevented with timely glucocorticoid (GC) treatment [5, 6]. This emphasizes the importance of preventing delay through early recognition, diagnosis, and treatment of GCA [7], which is challenging due to frequently occurring non-specific features [8, 9]. Traditionally, a temporal artery biopsy (TAB) is performed to diagnose GCA; however, this method has a low sensitivity, is invasive, and results are not instantly available. Even though treatment is often initiated before TAB results are available, unnecessary treatment should be avoided due to serious GC side effects. In recent years, rheumatology GCA fast-track clinics (FTCs) are introduced globally, contributing to a rapid diagnostic work-up after FTC referral including ultrasound (US) assessment of suspected GCA patients [10].

Despite FTC introduction, timely diagnosis remains challenging and diagnostic delay is not uncommon [11]. In a meta-analysis by Prior et al. [12] diagnostic delay was described; however, studies included in this meta-analysis did not primarily focus on diagnostic delay, only patients with GCA were described, and symptom duration before diagnosis was not studied. Therefore, more detailed information on reasons for delay in GCA suspected patients seen in an FTC is needed. The present study primarily investigates the time between onset of symptoms and first FTC visit in GCA suspected patients in a Dutch GCA FTC, and time to treatment initiation for those diagnosed with GCA. The study secondarily investigates the influence of GCA symptoms on time to first FTC visit in GCA suspected patients in an attempt to identify patients at risk for delay.

## Methods

### Design and subjects

This retrospective cohort study was conducted at the GCA FTC of the rheumatology outpatient department in Ziekenhuisgroep Twente (Hospital Group Twente), the Netherlands. Patients older than 50 years old suspected of GCA who first presented to the FTC from January 1, 2017 to October 1, 2019 were included. The study was conducted in accordance to the Helsinki code. The study protocol was approved by the METC Twente and was considered as not subject to the Medical Research Involving Human Subjects Act. Informed consent was waived by the METC Twente because of its retrospective nature. Clinical diagnosis at baseline by the treating rheumatologist with verification after 6 months was used to distinguish between patients with GCA (GCA+) and patients without GCA (GCA−).

### Data collection

The following data were collected: patient demographics, symptoms and clinical examination, laboratory parameters, results of diagnostic imaging and TAB if available, and dates regarding onset of symptoms, first visits to a general practitioner (GP) and/or specialists, referral to the FTC by a GP or another specialist, first FTC visit, and treatment initiation. Dates were collected using patient records and GP referral letters stored in electronic patient records. Generally, GCA indicated that treatment is not started by GPs in the Dutch healthcare system. Clinical GCA diagnosis was based on a combination of symptoms, clinical examination, inflammatory markers, and results of additional diagnostic imaging and/or TAB. GCA phenotype was established as C-GCA, LV-GCA, or overlapping C/LV-GCA. Patient data were collected from electronic health records. Castor study management system (Ciwit B.V., The Netherlands, version 2020.2.24) was used for data management.

### Definitions and time periods

Time periods between onset of symptoms, first GP visit, FTC referral, and first FTC visit were assessed. Time from onset of symptoms to treatment initiation could only be determined in GCA+ patients as GCA− patients did not receive GCA indicated treatment. This time period was defined as total delay. Cranial symptoms were defined as headache, jaw claudication, and/or scalp tenderness with or without visual symptoms [13]. Non-specific constitutional symptoms were defined as fever, weight loss, and/or fatigue [13].

### Statistical analysis

Mean values with standard deviation (SD) were used for normally distributed continuous variables, median values with interquartile ranges (IQR) for non-normally distributed variables. To compare independent groups, a Chi-square test, independent *T*-test, or Mann-Whitney *U* test was used when appropriate. A Kruskal-Wallis test and post hoc Mann-Whitney *U* tests with Holm-Bonferroni correction for multiple testing were performed to compare non-normally distributed variables amongst more than two independent groups. For each time period a complete case analysis was performed. A *p*-value of <0.05 was considered statistically significant. Statistical analyses were carried out in SPSS Inc. (Chicago, IL), version 24.

### Patient and public involvement statement

Patients were not involved in the design of this retrospective study.

## Results

### Baseline characteristics

In total, 205 patients with suspected GCA who visited the FTC between January 2017 and October 2019 were eligible for inclusion. In the total study population (*n* = 205), the mean age was 71.3 years (SD 10.7), and 55.1% (*n* = 113) were female. Baseline characteristics are summarized in Table 1. In the total study population, 29.8% (*n* = 61) patients were diagnosed with GCA (GCA+) and 70.2% (*n* = 144) patients were not diagnosed with GCA (GCA−) by the treating rheumatologist. In GCA+ patients, 67.2% (*n* = 41) had C-GCA, 13.1% (*n* = 8) had LV-GCA, and 19.7% (*n* = 12) had overlapping C/LV-GCA based on diagnosis by the treating physician. Diagnosis was confirmed and consistent with baseline diagnosis after 6 months in 58/61 GCA+ patients. Three GCA+ patients were deceased at 6 months (not GCA related). For these patients, a last observation carried forward approach was used.

**Table 1:** Baseline characteristics of all suspected GCA patients and stratified by GCA+ and GCA− patients

	Total study population (*n* = 205)	GCA+ (*n* = 61)	GCA− (*n* = 144)	*p*-Value
Gender, % (*n*) female	55.1 (113)	65.6 (40)	50.7 (73)	0.05
Age (years), mean (SD)	71.3 (10.7)	74.0 (9.3)	70.2 (11.0)	0.02
ESR^a^, median (IQR)	40.0 (23.0–85.5)	87.0 (55.0–103.8)	32.0 (14.0–59.0)	<0.001
*Missing*	*12*	*3*	*9*	
CRP^b^, median (IQR)	27.0 (4.0–70.3)	54.0 (40.5–110.5)	11.0 (2.0–51.3)	<0.001
*Missing*	*7*	*1*	*6*	
Polymyalgia rheumatica, % (*n*)	14.6 (30)	21.3 (13)	11.8 (17)	0.08
Cranial symptoms, % (*n*)	77.1 (158)	80.3 (49)	75.7 (109)	0.47
Headache	76.1 (156)	80.3 (49)	74.3 (107)	0.36
Jaw claudication	13.2 (27)	32.8 (20)	4.9 (7)	<0.001
Scalp tenderness	21.0 (43)	31.1 (19)	16.7 (24)	0.02
Constitutional symptoms, % (*n*)	47.8 (98)	59.0 (36)	43.1 (62)	0.04
Fever	6.8 (14)	9.8 (6)	5.6 (8)	0.36
Weight loss	23.9 (49)	29.5 (18)	21.5 (31)	0.22
Fatigue	31.7 (65)	36.1 (22)	29.9 (43)	0.38
Visual symptoms ^c^, % (*n*)	19.0 (39)	27.9 (17)	15.3 (22)	0.04
CVA or TIA^d^, % (*n*)	5.9 (12)	6.6 (4)	5.6 (8)	0.75
Diagnostics performed				
CDUS^e^, % (*n*)	96.6 (198)	96.7 (59)	96.5 (139)	1.0
TAB^f^, % (*n*)	30.7 (63)	59.0 (36)	18.8 (27)	<0.001
PET/CT^g^, % (*n*)	23.4 (48)	41.0 (25)	16.0 (23)	<0.001

^a^Erythrocyte Sedimentation Rate

^b^C-reactive protein

^c^Related to GCA (i.e. Anterior Ischemic Optic Neuropathy (AION), central retinal artery occlusion, diplopia)

^d^Cerebral Vascular Accidents or Transient Ischemic Attack

^e^Color Duplex Ultrasound

^f^Temporal artery biopsy

^g^18-FDG-Positron Emission Tomography/Computed Tomography

### Time to FTC visit in patients suspected of GCA

**Fig. 1:**
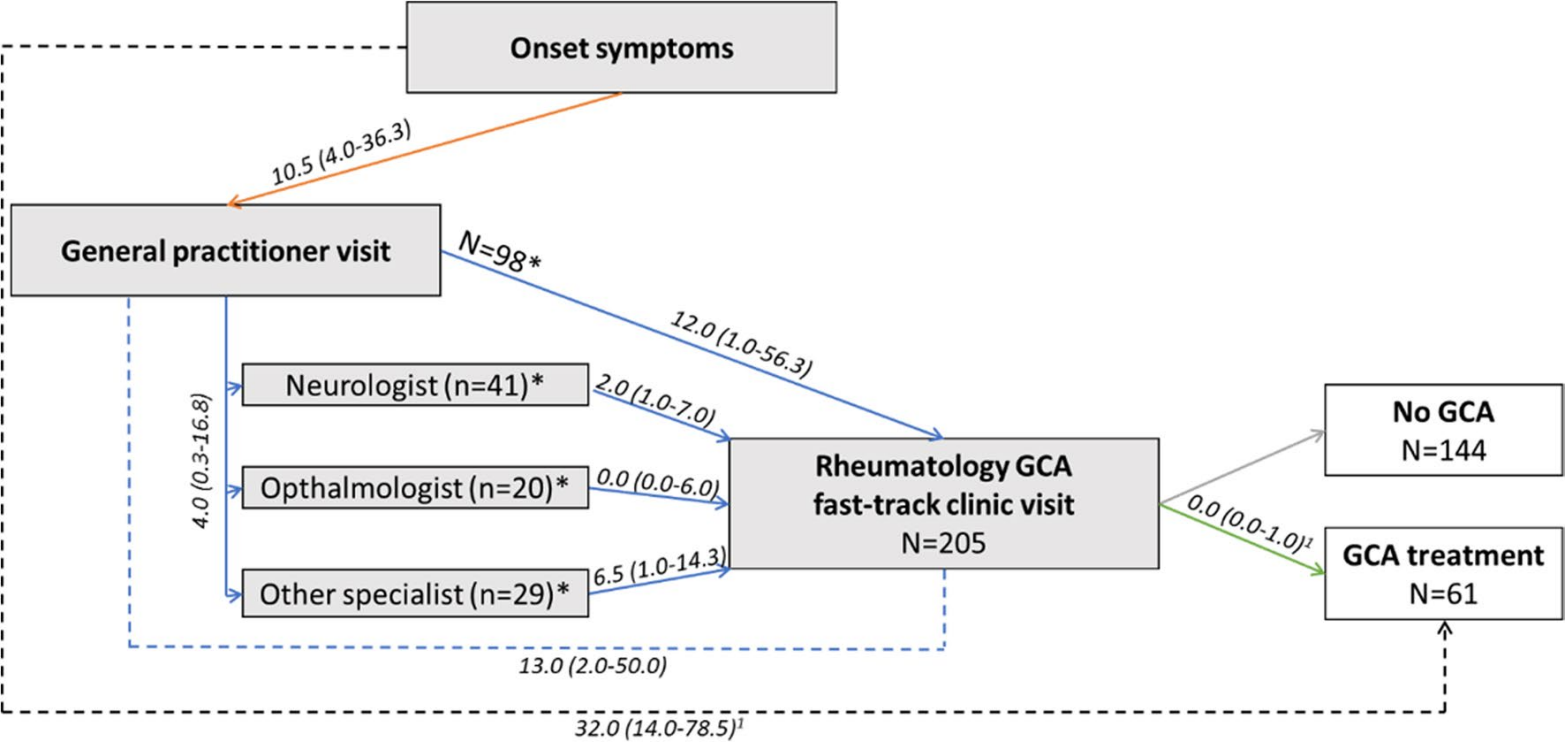
Types of delay and referral routes of patients with suspected giant cell arteritis in a Dutch peripheral hospital (*n* = 205). Median time periods are described in days (IQR). *For missing patients (*n* = 17), the referring physician was unknown. Other specialist were, e.g. geriatricians, internists, or otorhinolaryngologist. GCA: giant cell arteritis; ^1^could only be determined in GCA+ patients

Table 2A describes different components between onset of symptoms to first FTC visit in the total study population (*n* = 205). Median time between onset of symptoms and first consultation with a GP was 10.5 days (IQR 4.0–36.3). Median time between first GP visit and FTC referral was 10.0 days (IQR 1.0–47.5). In 98 patients, the GP referred directly to the FTC while 90 patients were first referred to a specialist other than a rheumatologist (Fig. 1). When directly referred to the FTC by a GP, median time between first GP visit and FTC referral was 9.5 days (IQR 0.8–51.3), and when referred to the FTC via another specialist this was 12.5 days (IQR 2.0–32.8) (*p* = 0.921). Patients were generally seen at the FTC within 1 day after referral (1.0 days [IQR 0.0–3.0]). In total, median time from onset of symptoms to first FTC visit was 31.0 days (IQR 13.0–108.8). No statistically significant differences were observed between GCA+ and GCA− patients in all time periods described above.

**Table 2:**
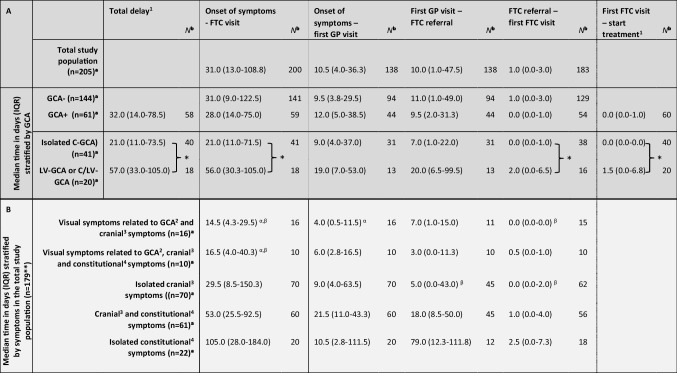
Time in steps between onset of symptoms and treatment initiation in GCA suspected patients

Time from onset of symptoms to GP visit, referral, FTC visit, and treatment in days stratified by (type of) GCA (A) and type of symptoms (B)

^a^Total number of patients per category is described

^b^Number of patients in particular time periods is described, which differs from the total number of patient per category

*Statistically significant (*p* < 0.05)

^α^Statistically significant difference with cranial and constitutional symptoms (Holm-Bonferroni)

^β^Statistically significant difference with isolated constitutional symptoms (Holm-Bonferroni)

^1^Calculation only possible in GCA+ patients

^2^Related to GCA (i.e. Anterior Ischemic Optic Neuropathy (AION), central retinal artery occlusion, diplopia)

^3^Headache, jaw claudication, and/or scalp tenderness

^4^Fever, weight loss, and/or fatigue

^**^Remaining patients presented with signs and symptoms that would lead to an irrelevant small patient group, for example with elevated CRP/ESR or visual symptoms without other cranial or constitutional symptoms

### Total delay in patients diagnosed with GCA

In addition, Table 2A describes median total delay from onset of symptoms to treatment initiation in GCA+ patients (*n* = 61), which was 32.0 days (IQR 14.0–78.5). GCA+ patients were seen at the FTC directly after referral (median 0.0 days [IQR 0.0–1.0]). At this first FTC visit, treatment generally was started instantly (median 0.0 days [IQR 0.0–1.0]). GCA+ patients were treated with high-dose GCs as indicated by the treating rheumatologist. For patients with isolated C-GCA (*n* = 41), median total delay was 21.0 days (IQR 11.0–73.5), while this was 57.0 days (IQR 33.0–105.0) in patients with LV-GCA or overlapping C/LV-GCA (*n* = 20) (*p* = 0.02).

### Time to first FTC visit stratified by type of symptoms

To identify patients at risk for longer delay, Table 2B shows time to first FTC visit stratified by type of symptoms in the total study population: isolated cranial symptoms with (*n* = 16) or without (*n* = 70) visual symptoms related to GCA, isolated constitutional symptoms (*n* = 22), and the combination of cranial and constitutional symptoms with (*n* = 10) or without (*n* = 61) visual symptoms related to GCA. Remaining patients (*n* = 26) presented with signs and/or symptoms less frequent in our patient group, such as an elevated CRP and/or ESR or isolated visual symptoms related to GCA. Time from onset of symptoms to first FTC visit was longest in the group of patients who presented with isolated constitutional symptoms (median 105.0 days [IQR 28.0–184.0]), while shortest for patients presenting with a combination of cranial and visual symptoms (median 14.5 days [IQR 4.3–29.5]) (*p* < 0.001).

## Discussion

This is the first study in the Netherlands that primarily investigated delay from onset of symptoms to first FTC visit in GCA suspected patients, including patients that were diagnosed with GCA as well as those not diagnosed with GCA. The median delay to first FTC visit was 31 days for the entire study population. No major differences in delay were observed between patients diagnosed with GCA or patients without GCA. We consider introduction of the FTC successful because all patients with suspected GCA were examined within a day after referral and GCA+ patients were treated with high-dose GCs immediately after diagnostic work-up. Also, our results showed that patients who presented with isolated constitutional symptoms had a longer delay.

Hospital Group Twente is the first general hospital in the Netherlands that introduced a GCA FTC with US in the standard diagnostic work-up. Compared to existing literature, delay towards diagnosis in our FTC is shorter. The meta-analysis by Prior et al. [12] described an average diagnostic delay of 9 weeks in GCA patients compared to a month in the present study, illustrating the success of our FTC. Furthermore, this meta-analysis reports a delay of 7.7 weeks for C-GCA patients and 17.6 weeks for LV-GCA patients [12]. This longer delay in LV-GCA was confirmed by our study. The study of Prior et al. also had some important limitations. First, delay was described as a secondary outcome measure and little information was available about how the information concerning delay was obtained. Also, the time frame covered in their study was from 1950 to 2013, in which disease awareness, knowledge, and diagnostic methods differed from today. In our study, we primarily investigated delay and incorporated different time periods within the referral and diagnostic progress in patients with suspected GCA who visited our FTC in more recent years. It has been reported that the introduction of FTCs may lead to a significant reduction in vision loss, mainly due to a shorter time period towards diagnosis and thereby early initiation of treatment [10, 14]. In line, we also previously described a case with a significant delay in diagnosis leading to iCVA, highlighting the importance of decreasing delay in GCA diagnosis [11]. Due to the small number of severe ischemic complications in the present study, analysis related to symptom duration and complications was not relevant.

In this study, patients were generally seen and treated with high-dose GCs within 1 day after referral to the FTC. Remaining delay was however considerable, and can either be attributed to the fact that patients are often unaware of the severity of their symptoms and therefore do not seek medical attention [15], or GPs might not recognize GCA at first presentation due to its generic symptoms and low incidence [16]. Therefore, to reduce total delay, increased awareness of signs and symptoms, mainly alarming symptoms such as sudden vision loss, is needed. In our cohort, around 30–40% of referrals with suspected GCA were diagnosed with GCA in our FTC. Education of the elderly population and GPs and classification of patients using pre-test probability tools may be helpful in increasing this percentage of positive diagnoses after referral in the future [17, 18].

Although a retrospective design can be a limitation, it had major advantages for this study. First, it allowed us to include a substantial number of GCA patients despite a low incidence. Furthermore, the electronic health records in our hospital contain elaborate information of GCA suspected patients including GP referral, which allowed us to study different aspects of delay within the referral process of a GCA FTC. Inevitably, there were missing data in each time period studied. As data were collected for healthcare purposes and missing data are therefore probably at random, minimal bias is expected. We did not exclude patients when data were missing to optimally use the available data, meaning that the number of patients used can differ per time period.

To conclude, this study shows that delay between onset of symptoms and FTC referral in GCA suspected patients still occurs despite a successfully implemented FTC. Patients with constitutional symptoms and extracranial manifestations had an increased delay compared to those with cranial and/or visual symptoms, who had already a delay of more than 2 weeks. Timely diagnosis and treatment remains crucial to prevent severe complications [7]. To reduce delay, early recognition of GCA-related symptoms is needed. Interventions at patient level and education of referring physicians — including the use of pre-test probability scores — could aid in raising awareness of the urgency of FTC assessment for GCA suspected patients [17–19].

## Data Availability

The data underlying this article will be shared on reasonable request to the corresponding author.

## References

[1] Ball EL, Walsh SR, Tang TY, Gohil R, Clarke JMF (2010). Role of ultrasonography in the diagnosis of temporal arteritis. Br J Surg.

[2] Li KJ, Semenov D, Turk M, Pope J (2021). A meta-analysis of the epidemiology of giant cell arteritis across time and space. Arthritis Res Ther.

[3] Salvarani C, Cantini F, Boiardi L, Hunder GG (2002). Polymyalgia rheumatica and giant-cell arteritis. N Engl J Med.

[4] Dejaco C, Duftner C, Buttgereit F, Matteson EL, Dasgupta B (2017). The spectrum of giant cell arteritis and polymyalgia rheumatica: revisiting the concept of the disease. Rheumatology (Oxford).

[5] Cho HJ, Bloomberg J, Nichols J (2017). Giant cell arteritis. Dis Mon.

[6] Liozon E, Boutros-Toni F, Ly K, Loustaud-Ratti V, Soria P, Vidal E (2003). Silent, or masked, giant cell arteritis is associated with a strong inflammatory response and a benign short term course. J Rheumatol.

[7] Baig IF, Pascoe AR, Kini A, Lee AG. Giant cell arteritis: early diagnosis is key. Eye Brain [Internet] 2019 17;11:1–12. 10.2147/EB.S170388 PMC6340646 30697092

[8] Ezeonyeji AN, Borg FA, Dasgupta B (2011). Delays in recognition and management of giant cell arteritis: results from a retrospective audit. Clin Rheumatol.

[9] Ponte C, Águeda AF, Luqmani RA (2018). Clinical features and structured clinical evaluation of vasculitis. Best Pract Res Clin Rheumatol.

[10] Diamantopoulos AP, Haugeberg G, Lindland A, Myklebust G (2016). The fast-track ultrasound clinic for early diagnosis of giant cell arteritis significantly reduces permanent visual impairment: towards a more effective strategy to improve clinical outcome in giant cell arteritis?. Rheumatology.

[11] van Nieuwland M, Boumans D, Plas GJJ, Vijlbrief OD, Alves C (2021). A tale of diagnostic delay with detrimental consequences: illustrating the challenging nature of diagnosing giant cell arteritis. Eur J case reports Intern Med.

[12] Prior JA, Ranjbar H, Belcher J, Mackie SL, Helliwell T, Liddle J (2017). Diagnostic delay for giant cell arteritis — a systematic review and meta-analysis. BMC Med.

[13] González-Gay MÁ, Ortego-Jurado M, Ercole L, Ortego-Centeno N (2019). Giant cell arteritis: is the clinical spectrum of the disease changing?. BMC Geriatr.

[14] Patil P, Williams M, Maw WW, Achilleos K, Elsideeg S, Dejaco C (2015). Fast track pathway reduces sight loss in giant cell arteritis: results of a longitudinal observational cohort study. Clin Exp Rheumatol.

[15] Ristvedt SL, Trinkaus KM (2005). Psychological factors related to delay in consultation for cancer symptoms. Psychooncology.

[16] Helliwell T, Muller S, Hider SL, Prior JA, Richardson JC, Mallen CD (2018). Challenges of diagnosis and management of giant cell arteritis in general practice: a multimethods study. BMJ Open.

[17] Laskou F, Coath F, Mackie SL, Banerjee S, Aung T, Dasgupta B (2019). A probability score to aid the diagnosis of suspected giant cell arteritis. Clin Exp Rheumatol.

[18] Neuman LM, van Nieuwland M, Vermeer M, Boumans D, Colin EM, Alves C (2021). External validation of the giant cell arteritis probability score in the Netherlands. Clin Exp Rheumatol.

[19] Gaspoz JM, Unger PF, Urban P, Chevrolet JC, Rutishauser W, Lovis C (1996). Impact of a public campaign on pre-hospital delay in patients reporting chest pain. Heart.

